# Transfer learning-enhanced CNN-GRU-attention model for knee joint torque prediction

**DOI:** 10.3389/fbioe.2025.1530950

**Published:** 2025-03-03

**Authors:** Hao Xie, Yingpeng Wang, Tingting Liu, Songhua Yan, Jizhou Zeng, Kuan Zhang

**Affiliations:** ^1^ School of Biomedical Engineering, Capital Medical University, Beijing, China; ^2^ Department of Rehabilitation, Beijing Rehabilitation Hospital, Capital Medical University, Beijing, China; ^3^ Department of Orthopedics, Beijing Luhe Hospital, Capital Medical University, Beijing, China

**Keywords:** knee joint torque, convolutional neural network, transfer learning, neuromusculoskeletal model, muscle force

## Abstract

**Introduction:**

Accurate prediction of joint torque is critical for preventing injury by providing precise insights into the forces acting on joints during activities. Traditional approaches, including inverse dynamics, EMG-driven neuromusculoskeletal (NMS) models, and standard machine learning methods, typically use surface EMG (sEMG) signals and kinematic data. However, these methods often struggle to reveal the complex, non-linear relationship between muscle activation and joint motion, particularly with complex or unfamiliar movements. The generalization of joint torque estimation models across different individuals faces a significant challenge, as feature transferability tends to decline in higher, task-specific layers, reducing model performance.

**Methods:**

In this study, we proposed a CNN-GRU-Attention neural network model combining a neuromusculoskeletal (NMS) solver-informed (hybrid-CNN) augmented with transfer learning, designed to predict knee joint torque with higher accuracy. The neural network was trained using EMG signals, joint angles, and muscle forces as inputs to predict knee joint torque in different activities, and the predictive performance of the model was evaluated both within and between subjects. Additionally, we have developed a transfer learning method in the inter-subject model, which improved the accuracy of knee torque prediction by transferring the learning knowledge of previous participants to new participants.

**Results:**

Our results showed that the hybrid-CNN model can predict knee joint torque within subjects with a significantly lower error (root mean square error ≤0.16 Nm/kg). A transfer learning technique was adopted in the inter-subject tests to significantly improve the generalizability with a lower error (root mean square error ≤0.14 Nm/kg).

**Conclusion:**

The transfer learning-enhanced CNN-GRU-Attention with the NMS model shows great potential in the prediction of knee joint torque.

## 1 Introduction

Accurate estimation of knee joint torque is important not only for understanding joint function but also for developing effective rehabilitation strategies and preventing injuries. Electromyography (EMG) signals provide insight into the electrical activity of muscles during contraction, which is intrinsically linked to the force produced by these muscles. Muscle force subsequently contributes to joint torque via the biomechanical leverage inherent in the musculoskeletal system, establishing EMG as a critical indicator of torque generation (Prilutsky and Gregor, 2000). Torque reflects the forces exerted by the surrounding muscles, making it a key factor in both athletic performance and the progression of joint conditions such as osteoarthritis (McErlain-Naylor et al., 2021; Zaman et al., 2022), evaluation of surgical outcomes (Berning et al., 2021) and design of exoskeletons or prostheses (Sartori et al., 2018; Yao et al., 2018; Pizzolato et al., 2019). Traditionally, the accurate measurement of knee joint torque has required specialized equipment, making it difficult to achieve real-time applications. Thus, predicting knee joint torque using electromyography (EMG) signals and advanced computational models has become an attractive solution.

Current methods for estimating knee joint torque mainly fall into two categories: EMG-driven neuromusculoskeletal (NMS) modeling and deep learning techniques. EMG-driven NMS models aim to capture the complex interactions among muscles, tendons, and joints by relating muscle activation signals to joint torque. While these models are highly informative, their application often involves intricate calibration procedures and the precise determination of numerous physiological parameters, such as muscle dynamics and kinematic variables, making their implementation both intricate and time-consuming. (Jung et al., 2022; Zhang et al., 2022; Zhao et al., 2023). The NMS model must first be calibrated through individual experiments to obtain personalized parameters, such as the optimal fiber length, optimal feather angle, maximum isometric contraction force, and tendon relaxation length before the personalized model can be used to estimate muscle force and joint torque (Serrancolí et al., 2016; Ao et al., 2022; Ao et al., 2023). Therefore, it is expected that the NMS model is lack of generalization for inter-subject predictions. This complexity can make them time-consuming to set up, limiting their practicality for real-time applications in clinical settings.

To address the time-consuming issues of physics-based musculoskeletal models, data-driven models have also been popular. These models can effectively capture nonlinear relationships between inputs such as joint angles and EMG signals, and outputs such as joint torques. Several studies used neural networks for the prediction of knee flexion angles or torques in able-bodied subjects (Dzulkifli et al., 2018; Xu et al., 2018; Hajian et al., 2021; Zhang et al., 2021; Schulte et al., 2022). Huang et al. (2019) proposed a deep-recurrent neural network for the prediction of knee joint angles in real-time. The model used EMG signals together with inertial data from different activities and reported a root mean squared error of 2.93° over a range of approximately 60° (4.9% error). Gautam et al. (2020) used a Long-term Recurrent Convolution Network to classify movements and predict their corresponding knee joint angles, based on EMG. They reported an average mean absolute error of 8.1% in the knee angle prediction of healthy subjects. Zhang et al. (2021) developed an artificial neural network for the prediction of ankle torque from EMG. Root mean squared error (RMSE) values in a range of approximately 1.5 Nm/kg were found for ankle plantar- and dorsiflexion. All these studies indicate that machine learning can be a valuable tool in predicting knee torque or knee angle. However, machine learning models do not account for the underlying mechanisms that link EMG signals to torque generation.

To address this issue, we propose a hybrid modeling approach that combines Convolutional Neural Networks (CNNs), Gated Recurrent Units (GRUs) and Attention mechanism with the NMS model. This innovative framework seeks to integrate the strengths of both NMS modeling and deep learning models, offering a more effective tool for estimating knee joint torque. The CNN component excels in extracting features from time-series data, while the GRU is well-suited for modeling temporal dependencies in joint movements. The Attention mechanism enhances the model’s ability to focus on key time points and significant features, improving prediction accuracy. However, the effectiveness of generalized models often diminishes significantly when applied to novel, previously unseen data, highlighting their limitations in handling unfamiliar scenarios. A notable example of this phenomenon can be seen in the work of Su et al. who introduced a Long Short-Term Memory (LSTM) model aimed at forecasting gait trajectories and phases over several future time frames (Su and Gutierrez-Farewik, 2020). Their findings indicated a significant reduction in the model’s performance when subjected to inter-subject testing, highlighting the challenges associated with applying the model to unfamiliar data. In response to this issue, researchers have increasingly turned their attention to transfer learning techniques in recent years. Soleimani et al. proposed a transfer learning framework that outperformed in the inter-subject scenarios. Transfer learning has emerged as an effective technique that utilizes knowledge from previous tasks to address challenges such as small sample sizes (Wu et al., 2023). By leveraging pre-trained models with characteristic parameters, this approach enhances the generalization capabilities of LSTM models, improving their performance across diverse and dynamic scenarios.

For addressing two key challenges: 1) the lack of personalized information in traditional machine learning models; 2) the performance degradation when testing on unseen data, we have developed a hybrid deep learning model that integrates transfer learning, CNN-GRU-Attention, and a musculoskeletal model. The objectives of this study are: 1) to compare the accuracy of knee joint torque prediction using a deep learning model integrated with a calibrated musculoskeletal model versus a standard deep learning model; 2) to investigate whether incorporating transfer learning improves the prediction accuracy of the model across subjects.

## 2 Materials and methods

### 2.1 Experiment setup

**FIGURE 1:**
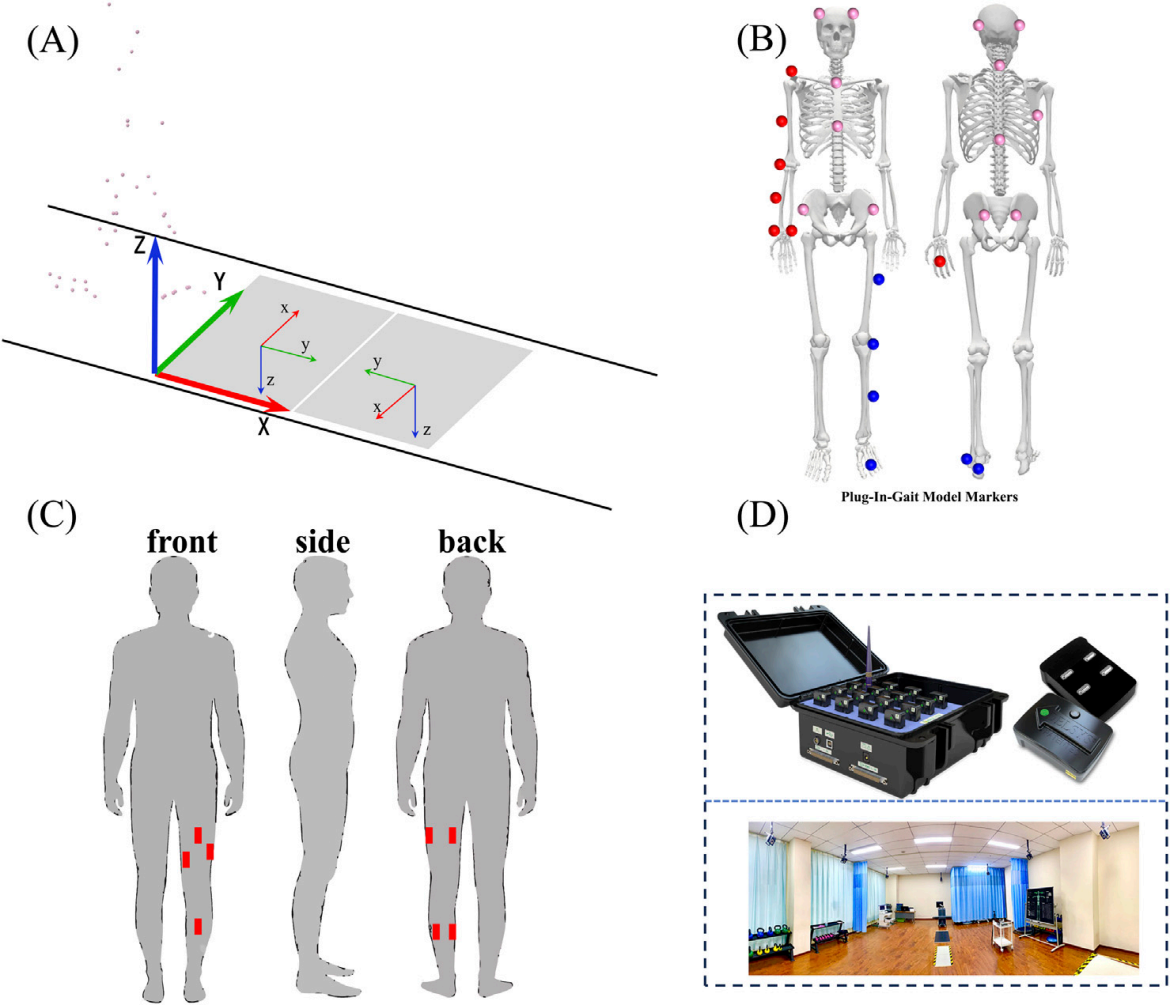
Shows marker and EMG sensor placement and plug-in-gait model based on captured marker positions. **(A)** represents the processing process of 3D motion capture data; **(B)** shows a model of reflective marker points used in plug-in gait; **(C)** shows the position of the EMG sensor in the acquisition process; **(D)** shows a 3D motion capture laboratory scene.

We used the GPOWER software (3.1.9.7) to calculate the sample size according to pre-experiment, and the sample size was calculated as 9. Ten healthy volunteers (age: 24 ± 3 years, height:1.74 ± 0.06 m, weight:70.9 ± 7.0 kg) were recruited for this study finally and the power with 10 subjects was 0.86. All participants provided informed consent prior to participation, and the study was approved by Capital Medical University. Participants were free of any musculoskeletal or neurological impairments, and none reported any recent injuries that could impact gait or knee function. Two movements were performed by each participant: isometric knee flexion-extension and walking at varying speeds. A 3D motion capture system (Vicon, Oxford, United Kingdom) and force plates (AMTI, USA) were used to record the kinematic and kinetic data of each task as shown in Figure 1.

Participants performed isometric contractions of the knee extensor and flexor muscles using a dynamometer (Biodex, System 4, Shirley, NY, USA). The knee joint was positioned at 30, 45, 60, 75 and 90-degree flexion angles, and participants were instructed to exert maximal voluntary isometric contraction (MVIC) for 5 s. A total of 3 trials were conducted with 3-minute rest intervals between trials. Torque output was recorded continuously to capture peak isometric strength and time-to-fatigue. We placed seven EMG electrodes on the medial gastrocnemius, lateral gastrocnemius, biceps femoris, semitendinosus, rectus femoris, vastus medialis, and vastus lateralis using a wireless surface EMG system at a 1,500 Hz sampling frequency in the two types of movements. Following the isometric tests, participants walked on an instrumented treadmill at three different speeds (0.8 m/s, 1.2 m/s, and 1.4 m/s). Each walking trial lasted 3 min, and participants were given a 2-minute rest between trials. 3D kinematic data were collected using the motion capture system, with reflective markers placed on the lower limbs according to the Plug-in Gait model. Ground reaction forces were recorded simultaneously with the force plates.

### 2.2 Data processing

The raw EMG data were band-pass filtered (20–450 Hz) to remove noise, followed by full-wave rectification. Signals were then normalized to MVIC values and a low-pass filter (6 Hz) was applied to obtain the envelope (Mantoan et al., 2015; Derrick et al., 2020). Kinematic data collected from the motion capture system during the gait trials were processed using OpenSim (v4.3), an open-source musculoskeletal modeling software (Pizzolato et al., 2016). Reflective markers were placed on the lower limbs according to the Plug-in Gait model. Inverse kinematics (IK) were performed in OpenSim to compute joint angles based on the 3D marker trajectories. Using inverse dynamics (ID), the net joint moments at the knee were calculated by combining the kinematic data with the ground reaction forces recorded from force plates. The muscle force used in this study is calculated using OpenSim’s Computed Muscle Control (CMC) tool.

### 2.3 Neural network architecture: CNN-GRU-attention model for torque prediction

**FIGURE 2:**
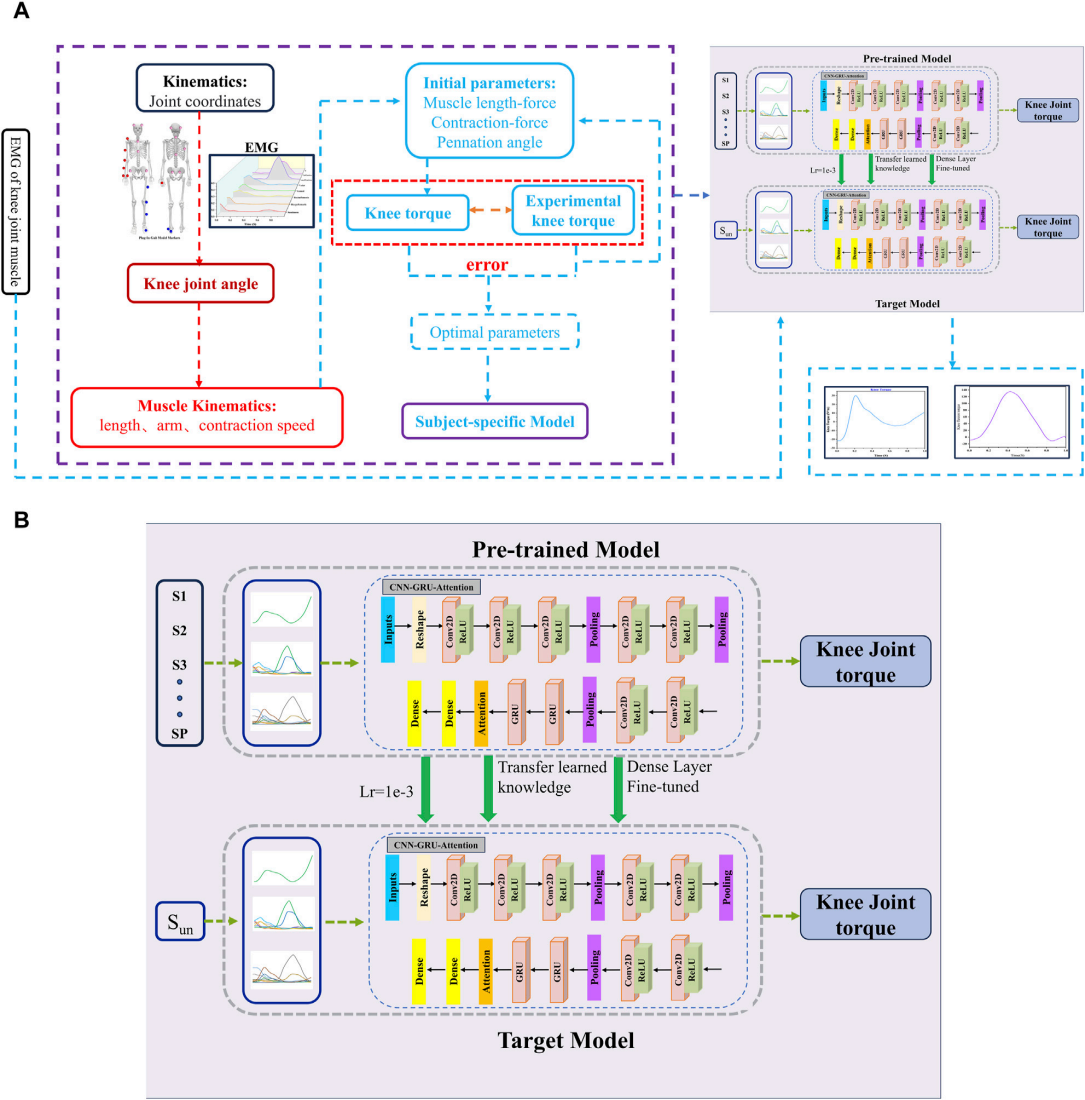
**(A)** Architecture of knee joint torque framework based on CNN-GRU-Attention network. For the hybrid-CNN model, computed muscle force through the physics-based calibrated NMS model was added. Panel **(B)** Network structure of CNN-GRU-Attention combined with transfer learning. We extracted layers from a pre-trained model and transferred it to the target model. In the target model, we tuned learning rate and retrained the last dense layer with data from the target subject.

In this study, a CNN-GRU-Attention model was established to predict knee joint torque. As shown in Figure 2, the model consists of A CNN, GRU and attention mechanism to perform regression on EMG and angle time series data. Firstly, CNN extracts feature from each input variable capturing local characteristics. GRU then captures long-term dependencies in the data. Finally, the attention module weights and sums the importance of input variables to enhance prediction performance. The model maps the features to the output variable space through dense layers. The personalized model used in this study is derived from previous work that employed an EMG-driven musculoskeletal model to calculate knee torque and reduce experimental measurement errors through an optimization algorithm.

#### 2.3.1 Convolutional neural networks (CNN)

The CNN is designed to extract spatial features from the time-series EMG signals, which are structured as 2D arrays where one dimension represents time and the other represents multiple EMG channels. The CNN architecture consists of several 2D convolutional layers with 3 × 3 convolution kernels. These kernels slide across the EMG data, detecting localized patterns related to muscle activation that are crucial for predicting joint torque. Each convolutional layer is followed by a Rectified Linear Unit (ReLU) activation function, which introduces non-linearity to the model. This helps the CNN capture complex relationships between muscle activation signals and torque output. The ReLU function is defined as Equation 1:

$$ReLU=\max\left(0,x\right)$$ (1)

To further reduce the dimensionality of the feature maps while retaining important information, max-pooling layers with a 2 × 2 window size are incorporated after specific convolutional layers. Max-pooling selects the maximum value within each 2 × 2 region, effectively down sampling the feature maps and mitigating overfitting.

The CNN architecture consists of alternating 2D convolutional layers and max-pooling layers, allowing the model to progressively reduce spatial dimensions while capturing increasingly complex patterns in the EMG signals. The convolution operation for the input 2D array X is expressed as Equation 2:

$$f_{i,j}=ReLU\left(\sum_{m=1}^{3}\sum_{n=1}^{3}\omega_{m,n}\cdot x_{i+m-1,j+n-1}+b\right)$$ (2)

where ω_m,n_ represents the 3 × 3 kernel, x is the input data, and b is the bias term. The output f_i,j_ forms the resulting feature map at position (i, j).

#### 2.3.2 Gated recurrent unit (GRU)

**FIGURE 3:**
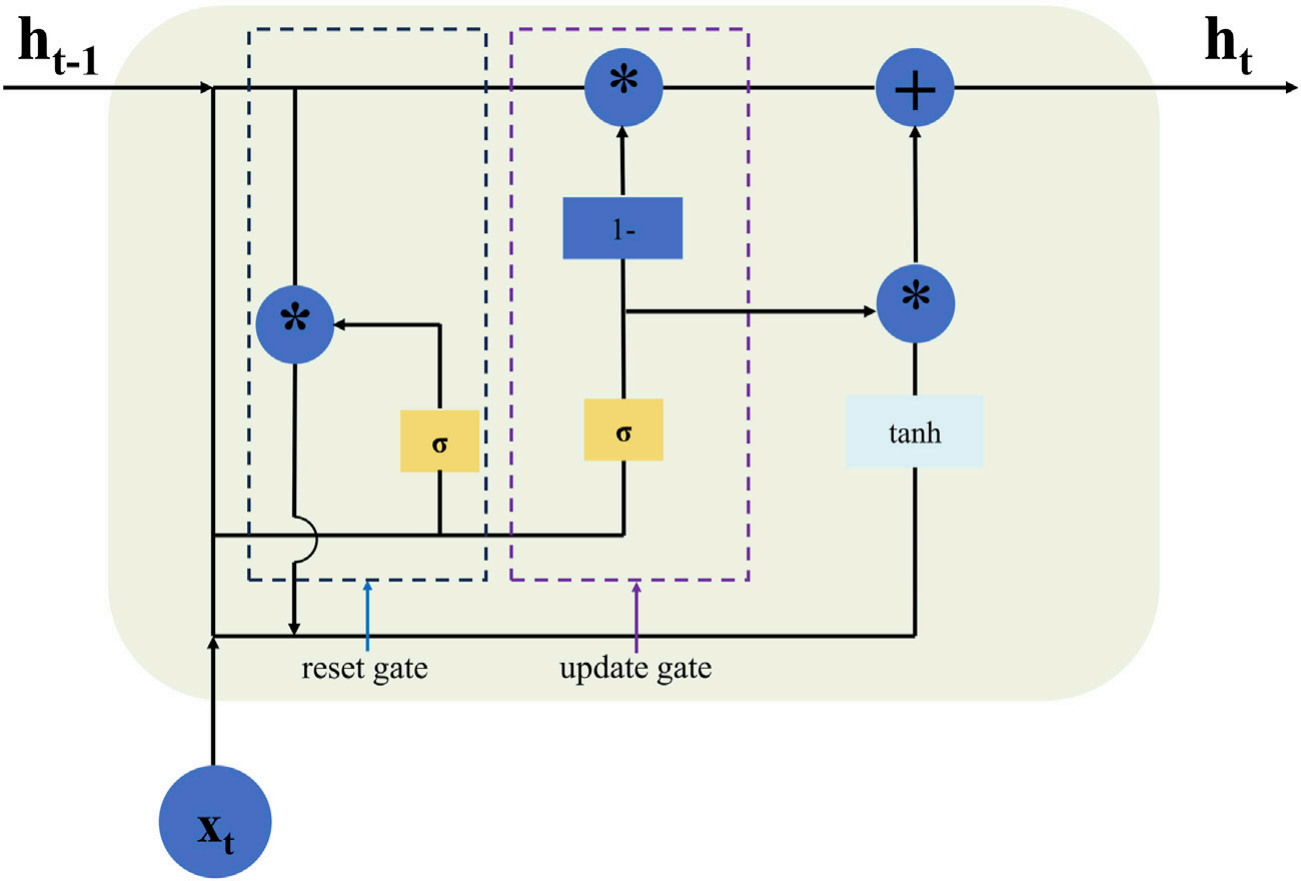
Shows the basic structure of GRU, the star (*) symbol denotes element-wise multiplication.

The gate recurrent unit (GRU) network is a simplified version of the long short-term memory (LSTM) network, sharing similar functionalities and gating mechanisms. It will acquire a faster training speed and make fewer errors than LSTM because of the fewer parameters considered (Xu et al., 2021). At the same time, owing to fewer parameters, the fitting effect of GRU is better than that of the LSTM with fewer original data (Choe et al., 2021). Figure 3 illustrates the structural relationship within the GRU unit at time t, where z_t_ and r_t_ represent the update and reset gate respectively which selectively remember or forget information, thereby mitigating the problems of vanishing and exploding gradients through the gating mechanism; h_t_ denotes the hidden state information of the GRU unit at this moment, while represents candidate state information. Nonlinear activation functions σ and tanh are applied to implement their respective functions in update gate z_t_ and reset gate r_t_ by utilizing previously hidden state information h_t-1_ and current input x_t_. The update gate determines the allocation ratio between hidden state information h_t_ at time t-1 and candidate state information at time t; a higher value retains more information from time t-1. Similarly, the reset gate controls the correlation degree between candidate state information at time t and hidden state information h_t-1_ at time t-1; a lower value leads to greater forgetting of past information at time t; a higher value retains more information from time t-1.

The calculation procedure of the forward pass in a GRU architecture can be expressed by the (Equations 3–6).

$$r_{t}=\sigma \left(\omega_{r}\cdot \left[h_{t-1},x_{t}\right]\right)$$ (3)

$$z_{t}=\sigma \left(\omega_{z}\cdot \left[h_{t-1},x_{t}\right]\right)$$ (4)

$$\tilde{h_{t}}=\tanh\left(\omega \cdot \left[r_{t}*h_{t-1},x_{t}\right]\right)$$ (5)

$$h_{t}=\left(1-z_{t}\right)*h_{t-1}+z_{t}*\tilde{h_{t}}$$ (6)

where, ω_r_, ω_z_ and ω are corresponding weight matrices; * represents matric multiplication.

#### 2.3.3 Attention mechanism

The attention mechanism is a widely used technique in the field of sequence data processing (Liu and Guo, 2019). Its basic idea is to dynamically assign weights, so as to selectively focus on specific parts of the input sequence. This method allows the model to deal with long-distance dependency problems more effectively, thereby improving the performance of sequence modeling. By calculating the similarity between any two positions, the attention mechanism can capture long-distance dependency relationships and is not limited by the sequence length. It is also suitable for processing various feature data and can flexibly adjust the weights of each feature to enhance the model’s performance and adaptability.

#### 2.3.4 Transfer learning

A transfer learning technique was employed to adapt the hybrid model for different participants in predicting knee joint torque. The pre-trained model on EMG data from certain subjects (S1, S2, …, Sp), was designed to capture general patterns in EMG relevant to torque prediction. For a new participant (St), only the final dense layer of the model was re-trained. With this approach, all layers of the pre-trained CNN-GRU -attention model, including convolutional, recurrent (GRU), and attention layers, were retained. However, the dense layer at the output, responsible for mapping the learned features to the torque prediction, was reinitialized and re-trained from scratch using the data from new subjects. The remaining layers were fine-tuned to adjust to the specific characteristics of the new subject while maintaining the knowledge acquired from previous subjects.

#### 2.3.5 Hyper parameters tuning for model

Hyperparameter tuning was conducted using a coarse-to-fine random search to optimize the CNN-GRU model for predicting knee joint torque from EMG signals (Bergstra and Bengio, 2012). The model was trained with a batch size of 512, using the Adam optimizer with an initial learning rate of 10^−3^, which was reduced to 10^−4^ during the transfer learning phase. The mean squared error (MSE) loss function was used to minimize prediction error. The tuning process explored a wide range of hyperparameters, including learning rates, CNN kernel sizes, and GRU units, followed by a refined search in promising regions. The model training was limited to 1000 epochs, after which optimization was stopped, and early stopping was applied to prevent overfitting based on validation loss.

### 2.4 Evaluation framework

**FIGURE 4:**
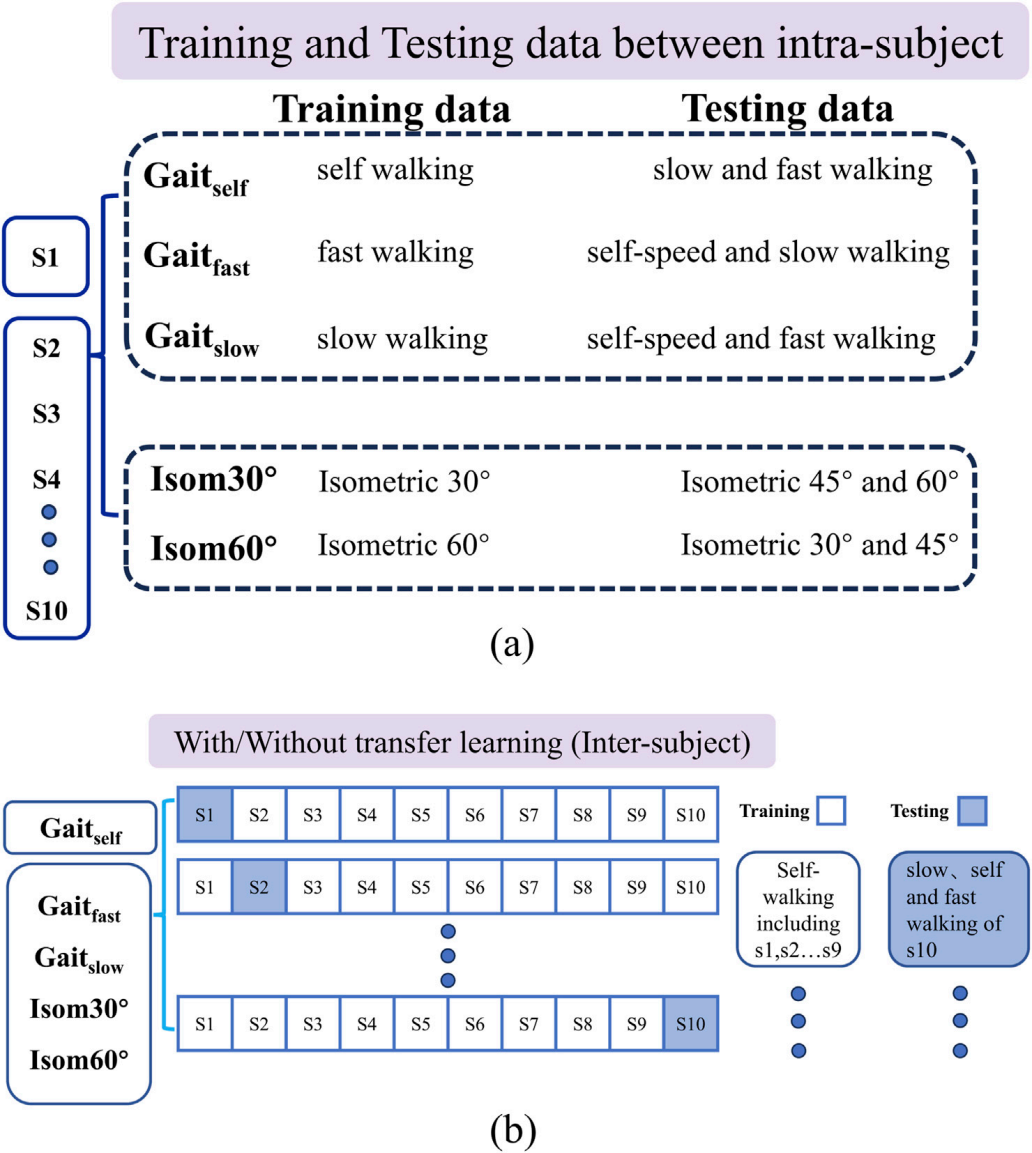
**(A)** The training and testing data hybrid-CNN model for five different cases in intra -subject prediction; **(B)** the training and testing data of hybrid-CNN model for five different cases in inter-subject prediction. Intra-subject prediction: For each case, different movements were used to train model. Models were trained by using data from each motion separately, and tested on the same type motions at different speeds, for each user individually. Inter-subject prediction without transfer learning: Models were trained for each movement of multiple subjects except one (leave-one-out cross-validation method), and then tested on the same type motions for the remaining new subject. Inter-subject prediction with transfer learning: Models were pre-trained for each movement on multiple users except one, and were shared to a new user with a common structure. We then re-trained models with data from the same motion of the new participant.

The study examines the accuracy of torque estimation by standard-CNN and hybrid-CNN models under two scenarios: intra-subject and inter-subject. In the inter-subject predictions, transfer learning is introduced to assess and compare the estimation accuracy of both models. For a detailed testing protocol, please refer to Figure 4.

#### 2.4.1 Intra-subject prediction

The model is trained on different movement variations within a single type of activity and tested on remaining variations. Specifically, two trials are used for training, while one trial serves as validation data.

#### 2.4.2 Without transfer learning

Both deep learning models were trained for each movement of multiple subjects except one (leave-one-out cross-validation method) and then tested on the same type of motions for the remaining new subject.

#### 2.4.3 With transfer learning

Both deep learning models were pre-trained for each movement on multiple subjects except one. We then re-trained the model using data from the same type of motion of the new subject and tested it on the remaining trials.

For each model, the prediction error is calculated as the root mean square error (RMSE) calculated as Equation 7 between predicted and measured joint torques, with the latter obtained via inverse dynamics. This RMSE is then normalized by body weight. To assess differences in prediction error between models, a paired-sample t-test is applied, with significance determined at the p < 0.05 level.

$$E_{RMS}=\sqrt{\frac{1}{N}\sum_{i=1}^{N}{\left(y_{p,i}-y_{i}\right)}^{2}}$$ (7)

## 3 Results

### 3.1 Intra-subject prediction performance

**FIGURE 5:**
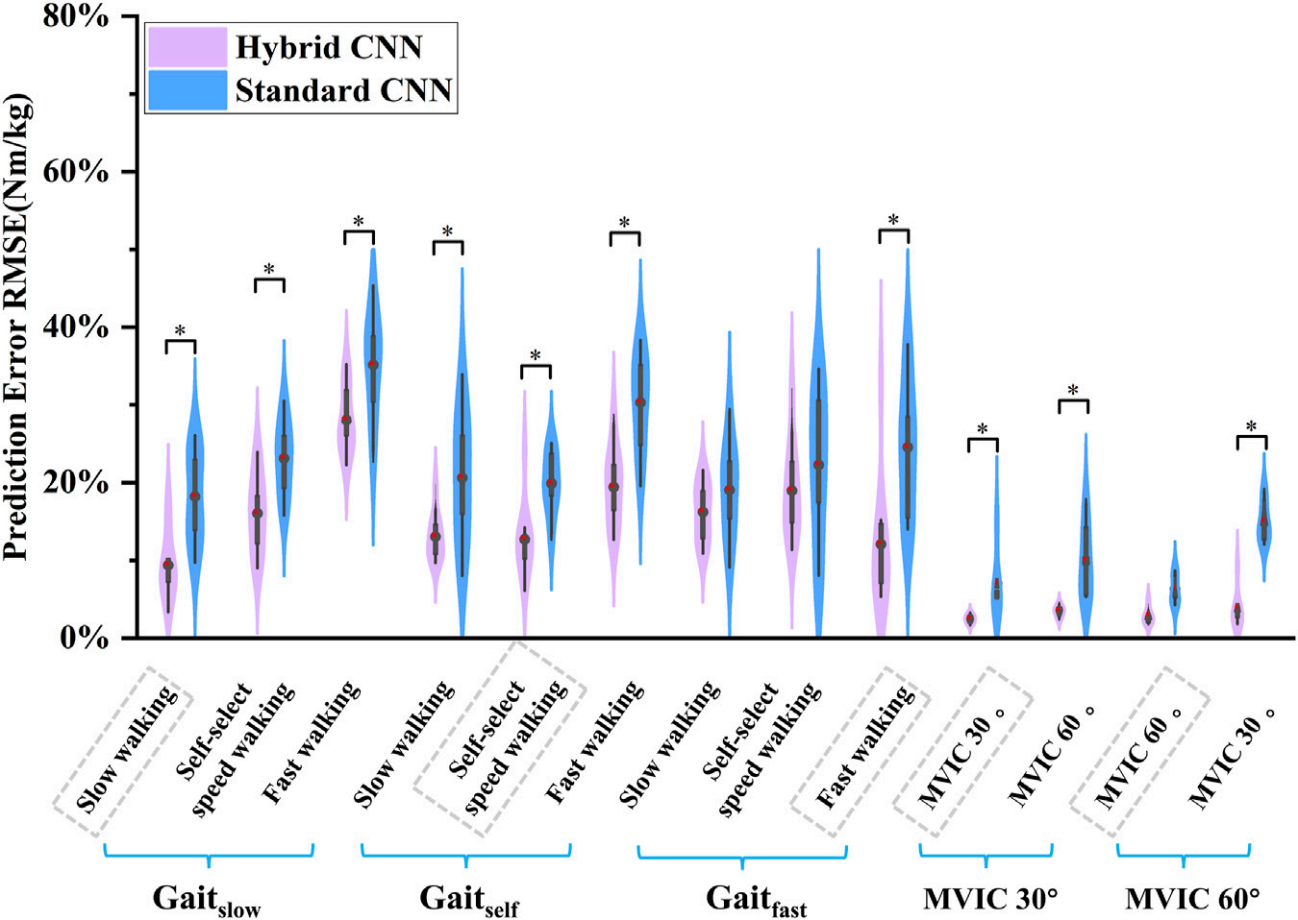
Violin plots depicting the distributions of RMSEs between predicted and measured joint torque (normalized by body mass) across subjects during five movements in the intra-subject scenario. ∗ indicates a significant difference between two cases.

**FIGURE 6:**
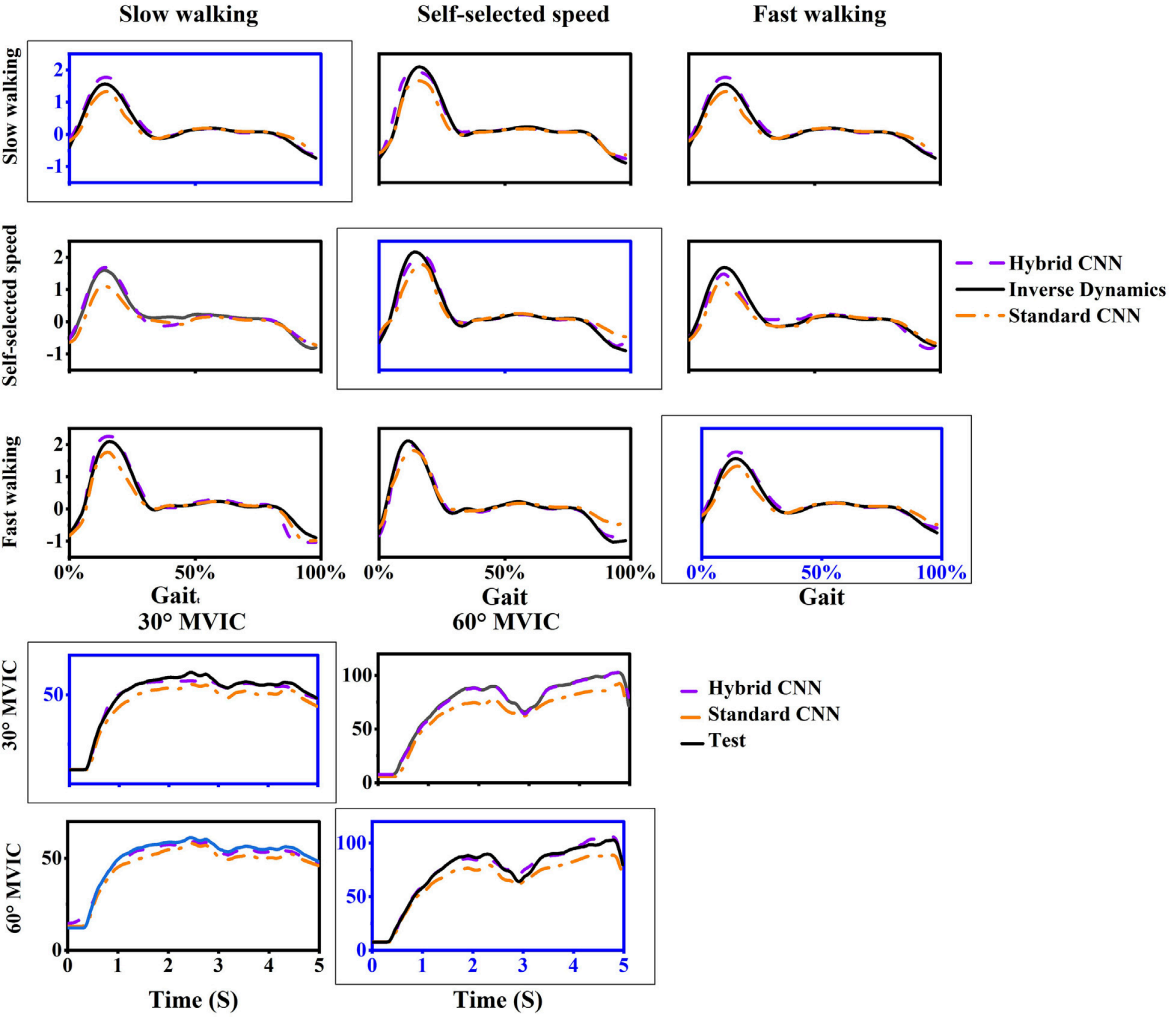
One example trial of measured (computed by inverse dynamics) and estimated joint torque via models during five movements in intra-subject scenario. For each case, the motion used as training data was framed with a dashed box and others are testing data.

Overall, compared to the standard-CNN model, the torque predictions from the hybrid-CNN model significantly demonstrated a superior agreement with the torque calculated through inverse dynamics at movement tests and smaller RMSE was observed in Figure 5 (hybrid-CNN: RMSE = 0.13Nm/kg, standard-CNN: RMSE = 0.21 Nm/kg). In all trained motions, the predicted accuracy by hybrid-CNN was significantly higher than that of the NMS. (slow walking: p = 0.004, self-selected speed walking: p = 0.004, fast walking: p < 0.0001, isometric knee extension 30°: p = 0.028, isometric knee extension 60°: p = 0.028). Compared to the standard CNN, the hybrid CNN did not always demonstrate superior prediction accuracy. It is worth noting that a worse predicted torque agreement with actual torque by the hybrid-CNN model and standard-CNN model was found in some tested motions, such as self-selected speed walking in Gait_fast_ case and fast walking in Gait_slow_ case (Figure 6).

### 3.2 Inter-subject prediction performance

**FIGURE 7:**
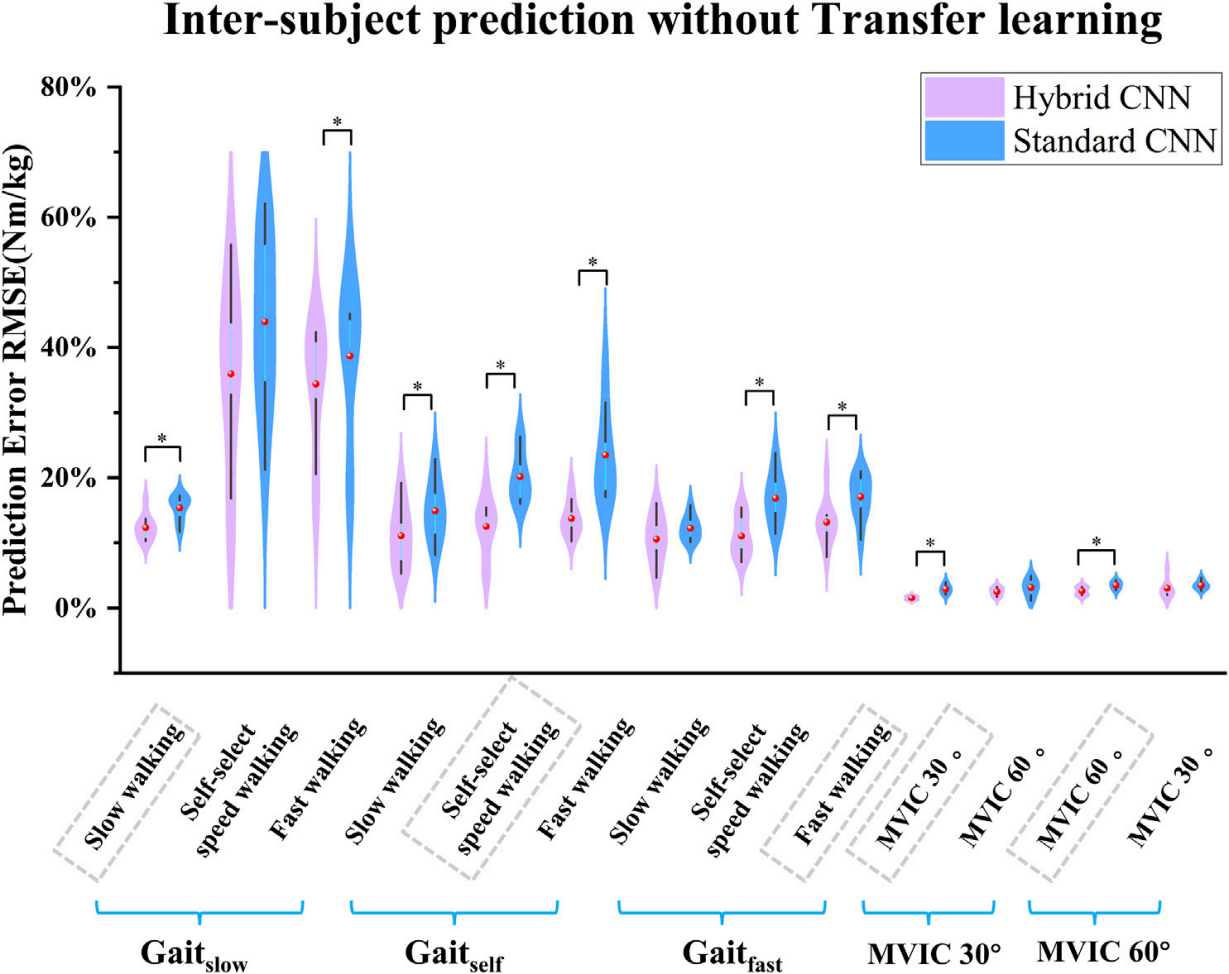
Violin plots depicting the distributions of RMSEs between predicted and measured joint torque without transfer learning (normalized by body mass) across subjects during five movements in inter-subject scenario. ∗ indicates a significant difference between two cases.

**FIGURE 8:**
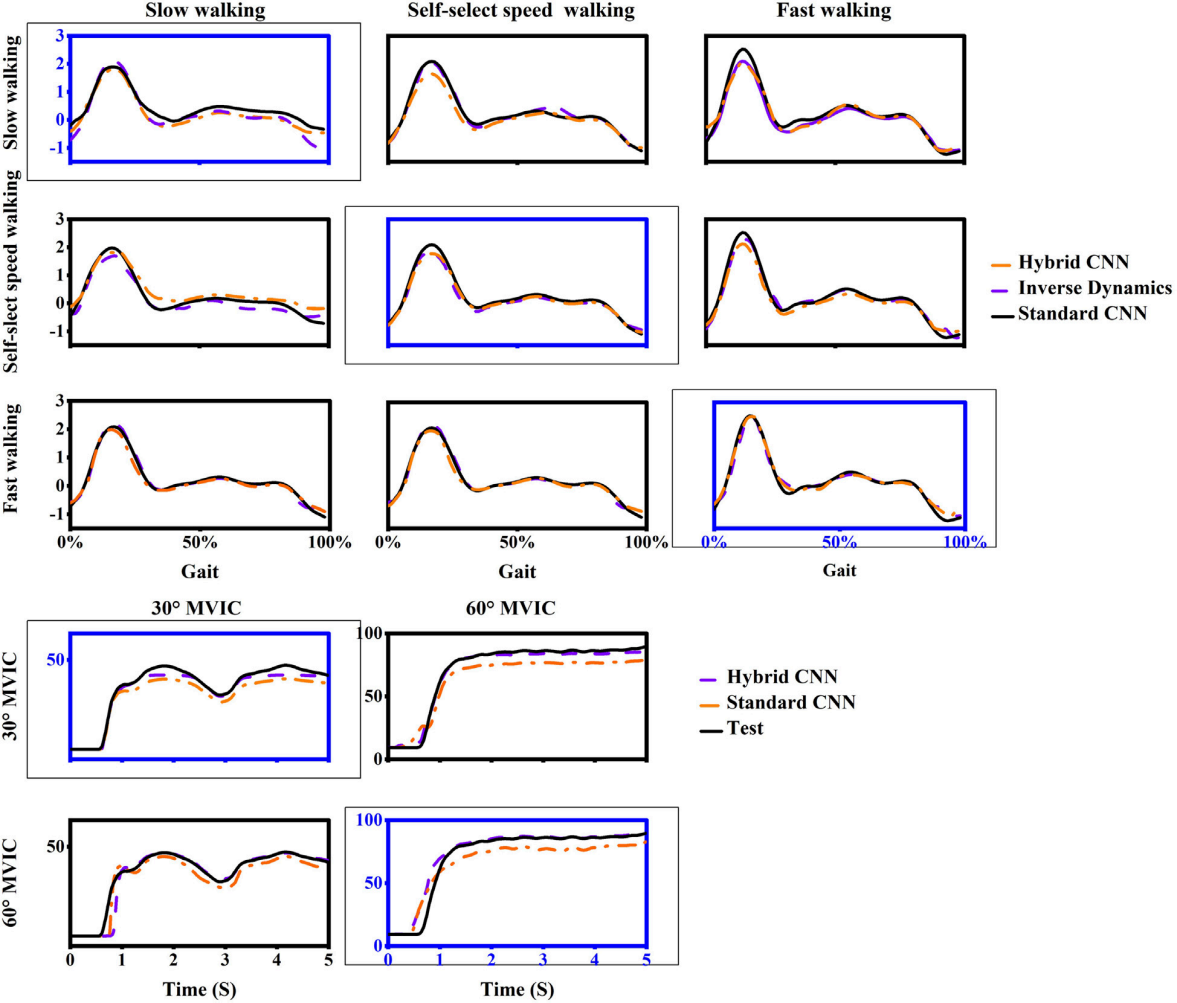
One example trial of measured (computed by inverse dynamics) and estimated joint torque via models without transfer learning during five movements in inter-subject scenario. For each case, the motion used as training data was framed with a dashed box and others are testing data.

Overall, the torque prediction error from the standard CNN model was higher than that from the hybrid CNN model before adopting transfer learning. It clearly showed that the standard CNN model performed worst in almost test scenarios (Figures 7, 8). In almost trained motions, the prediction accuracy by standard-CNN was significantly worse compared to hybrid CNN (slow walking: p = 0.038, self-selected speed walking: p = 0.011, fast walking: p = 0.025, isometric knee extension 30°: p = 0.028, isometric knee extension 60°: p = 0.028). In the tested movements, the standard CNN generally had worse prediction accuracy than the hybrid CNN model. The RMSE between predicted and calculated torque from inverse dynamics in the standard-CNN and hybrid-CNN model was 0.225 Nm/kg and 0.172 Nm.kg respectively.

**FIGURE 9:**
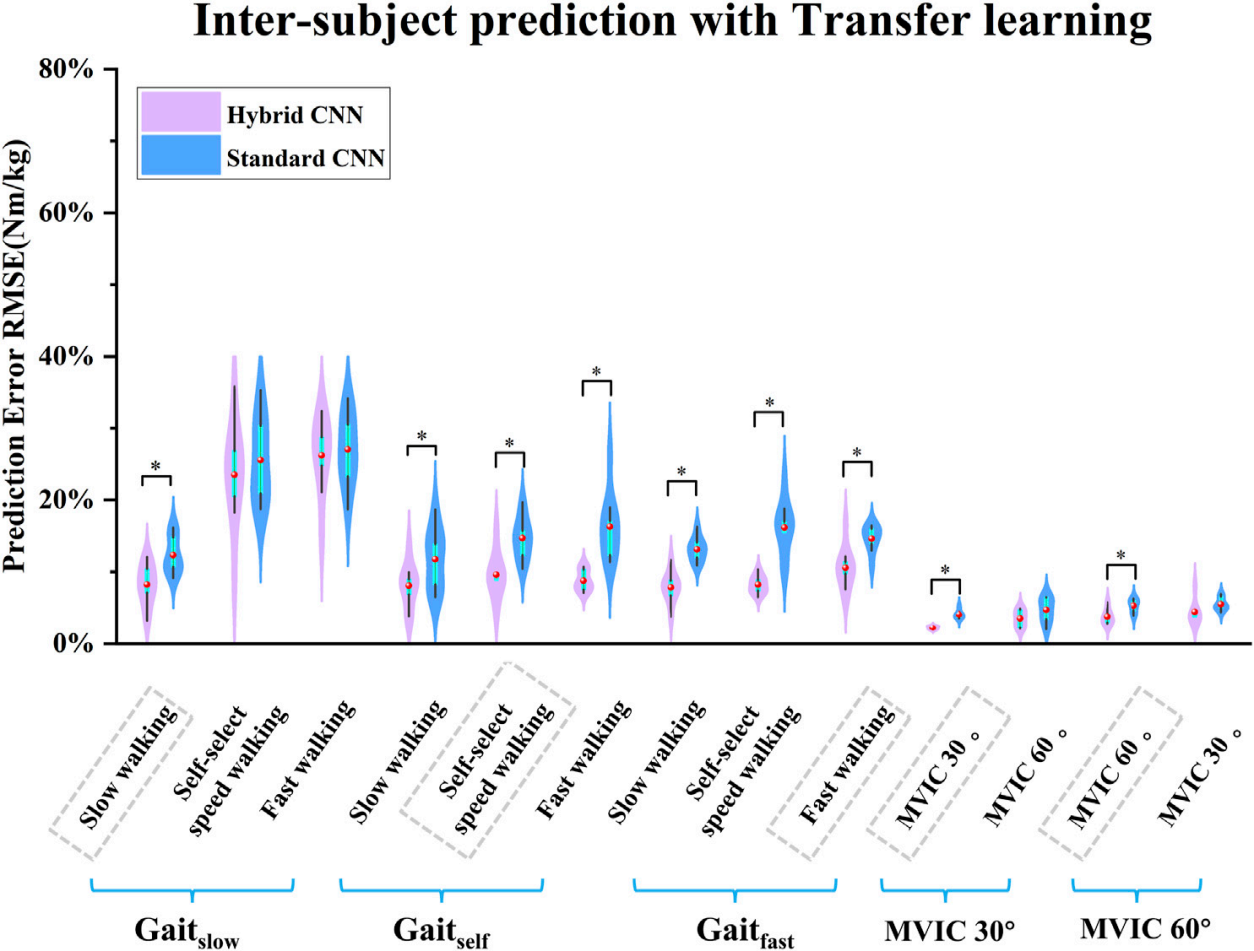
Violin plots depicting the distributions of RMSEs between predicted and measured joint torque with transfer learning (normalized by body mass) across subjects during five movements in inter-subject scenario. ∗ indicates a significant difference between two cases.

**FIGURE 10:**
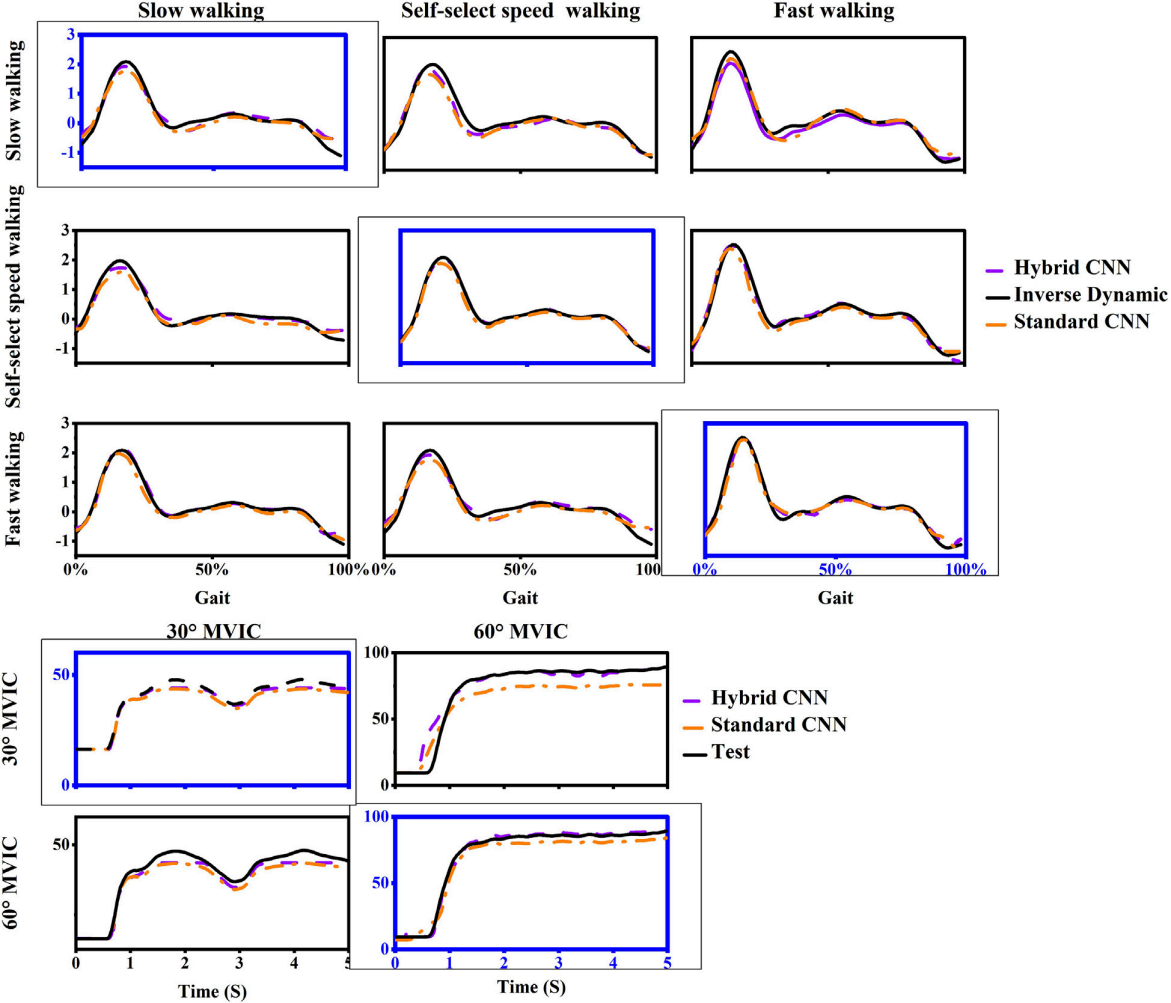
One example trial of measured (computed by inverse dynamics) and estimated joint torque via models with transfer learning during five movements in inter-subject scenario. For each case, the motion used as training data was framed with a dashed box and others are testing data.

After adopting transfer learning in both models, the predicted torque showed good agreement with the calculated and the RMSE had decreased wherein the standard-CNN had poorer accuracy in some movements compared to the hybrid-CNN model in terms of Figures 9, 10. The RMSE between predicted and calculated torque from inverse dynamics in the standard-CNN and hybrid-CNN model was respectively 0.168 Nm/kg and 0.12 Nm/kg and the prediction error significantly decreased 28.49% and 25.43% (p < 0.001) respectively.

## 4 Discussion

This study proposed a modeling method of transfer learning combined with convolutional neural networks-recurrent neural networks-attention mechanism (CNN-GRU-Attention) for the prediction of knee joint torque with much higher accuracy. This approach enhances model accuracy and generalization, particularly in predicting joint torque across diverse individuals and movement scenarios. This method extracts the common knowledge from a set of data through the pre-training of the network model, and extrapolates the knowledge to the target by fine-tuning (FT) of the network parameters, so as to quickly obtain a new adaptive model to realize the data feature transfer learning with small samples. The muscle forces calculated through the musculoskeletal model are entered into the model as features, and the performance between using (hybrid-CNN) and not using the muscle force model (standard-CNN) is compared. We observed a decline in torque prediction performance when extending the model to inter-subject scenarios. To address this issue, we implemented transfer learning, which significantly improved prediction accuracy and enhanced the generalization capability of the proposed model.

Gated recurrent unit (GRU) networks are frequently employed to predict joint torque and other sequence-based data in biomechanics. Serving as a model-free alternative to EMG-driven NMS models, GRUs enable direct mapping of EMG signals to joint torques, thus supporting real-time applications. Unlike NMS approaches, GRUs do not explicitly model physiological relationships, such as muscle excitation-activation dynamics, muscle force-length properties, and muscle-tendon kinematics at various joint angles. By contrast, NMS models are typically customized to individual subjects, using experimental data to refine parameters like optimal muscle fiber length and tendon slack length, thereby improving joint torque predictions on a subject-specific basis (Pizzolato et al., 2015; Hoang et al., 2018). Due to the individual calibration, NMS models are rarely evaluated for cross-subject generalizability. However, for situations requiring precise, subject-specific accuracy, NMS-based methods are often the preferred choice. Integrating muscle forces derived from musculoskeletal (MSK) models with EMG signals and joint angles as inputs to deep learning models significantly improves knee joint torque prediction over standard CNNs. As shown in Figure 5, the mean RMSE is 0.178 Nm/kg, 0.15 Nm/kg and 0.157 Nm/kg in the case of Gait_slow_, Gait_self_ and Gait_fast_ using hybrid-CNN. Also, the mean RMSE is 0.255 Nm/kg, 0.231 Nm/kg and 0.217 Nm/kg in the case of Gait_slow_, Gait_self_ and Gait_fast_ using standard-CNN. The prediction error decreased 18%, 35.06% and 27.64% respectively in intra-subject scenarios. This enhancement stems from incorporating physiologically meaningful data that better reflects the human musculoskeletal system. Muscle force serves as a crucial intermediate variable that bridges the complex relationship between muscle activation and joint dynamics, allowing the model to capture non-linear biomechanical interactions more effectively. Combining EMG and joint angles with MSK-derived muscle force provides a multi-source input approach, offering comprehensive insights into movement phases and muscle responses, thus reducing prediction errors. Moreover, muscle force data help the model adapt to inter-subject variability and unseen movement patterns, enhancing robustness against data distribution shifts.

As expected, when transfer learning was not adopted, inter-subject torque prediction performance was less accurate than that of intra-subject prediction, regardless of which hybrid-CNN model was used. Without transfer learning, both hybrid- and standard CNN were trained using data from previous experiences/subjects but none from the new subject. Therefore, it is to be expected that the torque prediction would be less accurate than the calculated from inverse dynamics. When transfer learning was implemented, the joint torque prediction performance was significantly improved in almost all cases as shown in Figure 9. When using the hybrid CNN model, the mean RMSE was 0.123 Nm/kg and 0.172 Nm/kg respectively with and without transfer learning. It can be concluded that the predictive error has significantly reduced by 28.49%. Similarly, when using the standard CNN model, the mean RMSE was respectively 0.168 Nm/kg and 0.225 Nm/kg with and without transfer learning. It can be concluded that the predictive error has witnessed a significant reduction of 25.33%. Transfer learning enhances adaptability across patient populations by enabling models to fine-tune efficiently on specific datasets, reducing the need for extensive data collection. Techniques such as model pruning and lightweight architectures ensure low-latency performance, critical for real-time tasks like prosthetic control, gait analysis, and rehabilitation monitoring. Additionally, transfer learning improves generalization to diverse clinical conditions, supporting applications such as fall risk prediction, post-surgical mobility assessment, and remote monitoring of chronic conditions.

Muscle coordination patterns can be expected to vary across subjects (Safavynia et al., 2011; Rugy et al., 2012), thus, the standard-CNN model may not have sufficient generalizability without information from a new subject in the training process, particularly when training data sets with other subjects are not rich enough. Transfer learning is a common approach in inter-subject cross-validation, improving the generalization of neural networks by transferring knowledge from one domain (previous subjects) to another (new participants) (Soleimani and Nazerfard, 2021). This approach effectively mitigates the decline in predictive accuracy observed when evaluating models on previously unseen data. A study by Kian et al. found that the effectiveness of EMG-driven NMS model calibration is task-dependent, suggesting the use of diverse tasks to optimize musculotendon and EMG-to-activation parameters (Kian et al., 2021). However, individuals with disabilities may struggle to perform a wide range of tasks. Transfer learning proves especially valuable when collecting large datasets from new subjects or movements is costly, time-intensive, or challenging, as in the case of motor disabilities. By incorporating transfer learning, joint torque prediction accuracy was significantly improved.

One limitation of this study lies in the relatively small sample size, which might restrain the diversity of the input data and, accordingly, the model’s capacity to capture a broader range of variability in biomechanical and physiological characteristics. Additionally, the participant group was confined to young adults, which precludes the exploration of how age-related factors, such as alterations in muscle strength or joint stiffness, could impact the model’s performance. Also, the accuracy of this model is verified only in different walking speeds and isometric test movements. Future studies should expand the dataset to include older adults, different movement such as jumping and running, pediatric populations, and individuals with musculoskeletal or neurological conditions. These diverse cohorts would allow us to assess the model’s robustness and adaptability across a broader range of biomechanical patterns, ultimately enhancing its clinical applicability.

## 5 Conclusion

This study developed an advanced hybrid neural network model that integrates biomechanical parameters from an NMS with CNNs to enhance knee joint torque prediction accuracy. The results reveal that the hybrid model surpasses the performance of standard-CNN in intra-subject assessments. This improvement is attributed to the inclusion of physiological variables, such as individualized muscle forces computed by the hybrid CNN, which act as essential intermediaries in knee torque estimation and boost prediction accuracy, especially when faced with new movement patterns. To address the challenges in inter-subject torque prediction, the study incorporates transfer learning into the NMS-informed CNN model. This approach significantly improves predictive accuracy for unseen movements, such as slower walking, notably during the late stance phase when peak knee extension torque occurs. The transfer learning-enhanced CNN-GRU-Attention with the NMS model outperforms both hybrid-CNN without transfer learning and standard-CNN significantly, and shows great potential in the prediction of knee joint torque.

## Data Availability

The original contributions presented in the study are included in the article/supplementary material, further inquiries can be directed to the corresponding author.
